# Isolation and Biochemical Characterization of *Apios* Tuber Lectin

**DOI:** 10.3390/molecules20010987

**Published:** 2015-01-09

**Authors:** Eri Kenmochi, Syed Rashel Kabir, Tomohisa Ogawa, Ryno Naude, Hiroaki Tateno, Jun Hirabayashi, Koji Muramoto

**Affiliations:** 1 Graduate School of Life Sciences, Tohoku University, Katahira 2-1-1, Aoba-ku, Sendai 980-8577, Japan; E-Mails: eri.kenmochi@gmail.com (E.K.); rashelkabir@ru.ac.bd (S.R.K.); ogawa@biochem.tohoku.ac.jp (T.O.); 2 Department of Biochemistry and Molecular Biology, University of Rajsahi, Rajshahi 6205, Bangladesh; 3 Department of Biochemistry and Microbiology, Nelson Mandela Metropolitan University, Port Elizabeth 6031, South Africa; E-Mail: ryno.naude@nmmu.ac.za; 4 National Institute of Advanced Industrial Science and Technology, 1-1-1 Umezono, Ibaraki 305-8568, Japan; E-Mails: h-tateno@aist.go.jp (H.T.); jun-hirabayashi@aist.go.jp (J.H.)

**Keywords:** *Apios americana*, lectin, American groundnut, legume lectin

## Abstract

*Apios* tuber lectin, named ATL, was isolated from *Apios americana* Medikus by two chromatography steps, hydrophobic chromatography and anion-exchange chromatography. The minimum concentration required for the hemagglutination activity toward rabbit erythrocytes of ATL was 4 μg/mL. ATL was composed of a homodimer of 28.4 kDa subunits. The amino acid sequence of ATL was similar to those of other legume lectins. The lectin showed moderate stability toward heating and acidic pH, and the binding affinity against several monosaccharides, such as D-glucosamine and D-galactosamine. ATL also bound to desialylated or agalactosylated glycoproteins such as asialo and agalacto transferrin. ATL decreased the transepithelial electrical resistance across human intestinal Caco-2 cell monolayers, suggesting the effect on the tight junction-mediated paracellular transport.

## 1. Introduction

Lectins are a class of proteins that recognize carbohydrate structures and bind carbohydrates specifically, and are found in a wide variety of plants, animals, and microorganisms [1]. At present, a few hundred plant lectins have been isolated and characterized in terms of their molecular structures, carbohydrate specificities, and biological properties [2]. Plant lectins have been classified into seven families; the legume lectins, the monocot mannose-binding lectins, the jacalin-related lectins, the Cucurbitaceae phloem lectins, the chitin-binding lectins composed of hevein domains, the amaranthin lectins, and the ribosome-inactivating proteins [3]. The functional roles of plant lectins are proposed to involve biological defense [3,4,5,6], mutualism for nitrogen fixation bacteria [7], and transportation/storage proteins in plant bodies [3].

Lectins are particularly abundant in the seeds of legumes, and account for approximately 10% of the soluble proteins of the seed extracts [8]. The legume lectin family is the largest in the plant lectin family. Legume lectins consist of two or more subunits of 25 to 30 kDa and show a wide variation of sugar-binding affinities, despite their high similarity of the amino acid sequences among the family members, *i.e.*, soybean lectin (SBA) has d-galactose (Gal)/*N*-acetyl-d-galactosamine (GalNAc) binding specificity, whereas concanavalin A (ConA) is d-mannose (Man)/d-glucose (Glc) specific.

Lectins in foodstuffs are considered to be food factors which affect the intestinal function by interacting with epidermal cells of small intestine [9]. In fact, some lectins have tolerance against heating, acidic pH and digestive enzymes, and maintain a significant activity in the small intestine [10,11]. For example, SBA retains 50% of its activity under the digestive conditions such as those found in the stomach and intestine. The digestive tract is therefore constantly exposed to the biologically active lectins contained in fresh and processed foods [3,10]. Since the epithelial surface of the intestine is extensively glycosylated, lectins interact with this surface and can induce physiological effects on humans and other animals, particularly when consumed in large quantities. We have shown that lectins contained in foodstuff had varying modulating effects on the transport system of human intestinal Caco-2 cell monolayers [12,13].

*Apios americana* Medikus, sometimes called American groundnut, is an edible tuberous lugume native to Eastern North America. The tubers are nutritious, with a high content of protein (12%–13%) [14]. The plant has been commercially farmed in the northern part of Japan since its introduction to Japan from North America in the 19th century. It has been reported that *Apios* tubers have blood pressure lowering effects and can improve lipid metabolism [15,16]. In fact, various bioactive molecules such as isoflavones have been reported in *Apios* tubers [17,18,19]. Furthermore, Zhang *et al.* reported the purification, characterization and cDNA cloning of a Bowman-Birk type trypsin inhibitor [20,21] and a lectin [22] from *Apios* tubers.

In this paper, we adopted a different approach to isolate a lectin from *Apios* tubers and characterized its biochemical properties in more detail to evaluate the lectin as a functional food factor. The lectin showed characteristic properties such as sugar binding specificity and decreased the transepithelial electrical resistance (TER) across human intestinal Caco-2 cell monolayers.

## 2. Results and Discussion

### 2.1. Isolation of Apios Tuber Lectin (ATL)

**Figure 1:**
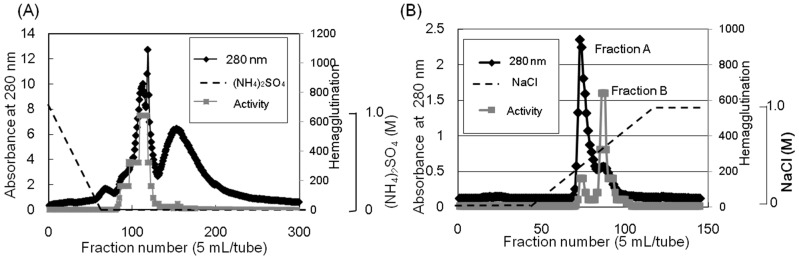
Purification of the *Apios* tuber lectin (ATL). (**A**) The protein fraction obtained by 40% saturated ammonium sulfate precipitation was separated by hydrophobic chromatography on a Toyopearl phenyl-650M column (2.8 × 16 cm) pre-equilibrated with 1.0 M ammonium sulfate in 50 mM Tris-HCl buffer (pH 8.0). Proteins were eluted with a decreasing linear gradient of ammonium sulfate (1.0–0 M) in the same buffer. Factions showing hemagglutination activity were collected and dialyzed against 50 mM Tris-HCl buffer (pH 8.0). (**B**) The dialysate was subjected to anion-exchange chromatography on a Toyopearl DEAE-650M column (2.8 × 32 cm) pre-equilibrated with 50 mM Tris-HCl (pH 8.0) and eluted with a linear gradient of NaCl (0 to 1.0 M) in the same buffer. Fractions A and B (ATL) were dialyzed against distilled water and lyophilized.

**Figure 2:**
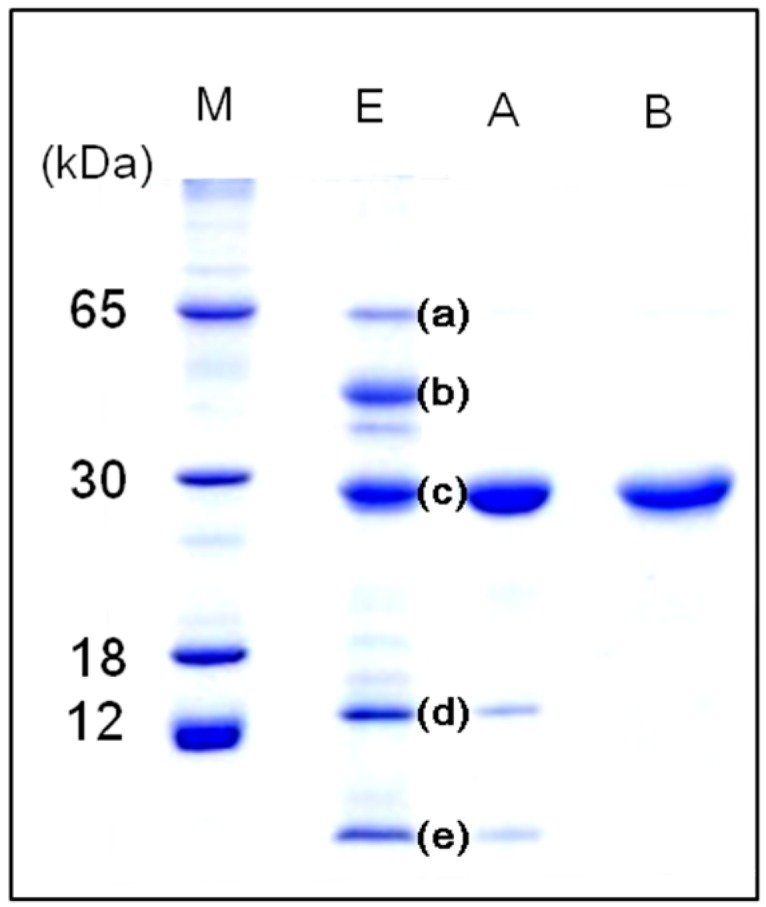
SDS-PAGE patterns of *Apios* tuber proteins in 15% acrylamide gel under reducing condition. Lanes M: Protein markers, E: *Apios* protein fraction obtained by ammonium sulfate precipitation, A: Fraction A; B: Fraction B (*Apios* tuber lectin).

**Figure 3:**
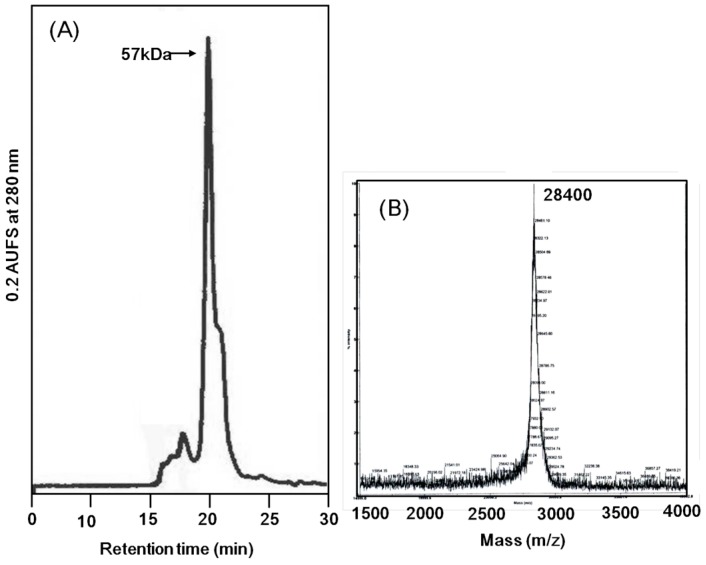
Molecular mass determination of *Apios* tuber lectin. (**A**) Size exclusion chromatography on a PC200S (N) column (5 μm, 7.8 × 300 nm) in 50 mM HEPES (pH 6.9) containing 0.25 M NaCl and 5 mM CaCl_2_ as the mobile phase. Flow rate: 0.8 mL/min; UV detection: 280 nm. (**B**) MALDI-TOF mass spectral analysis.

*Apios* tuber lectin was isolated by hydrophobic chromatography and anion exchange chromatography (Figure 1). Two major peaks, Fractions A and B, were obtained by anion exchange chromatography with yields of 240 mg and 60 mg from 200 g of the tubers, respectively (Table 1). Fraction B showed strong hemagglutination activity with the minimum concentration of 4 μg/mL required for the activity against rabbit erythrocytes. The isolated *Apios* tuber lectin (Fraction B), named ATL, gave a single protein band of 28 kDa during SDS-PAGE in the presence and absence of 2-mercaptethanol (Figure 2). Fraction A also gave a single band of 28 kDa during SDS-PAGE, but showed only marginal hemagglutination activity. As shown in Figure 2, ATL and Fraction A protein (band c) were major components in the extract of *Apios* tubers. The apparent molecular weight of ATL was estimated to be 57 kDa by size exclusion chromatography (Figure 3A). The MALDI-TOF mass spectrometry of ATL gave a peak of 28.4 kDa (Figure 3B). Fraction A protein showed a similar molecular mass. These results indicate that both ATL and Fraction A protein are composed of non-covalently bound homodimers.

**Table 1:** Summary of the purification of *Apios* tuber lectin (ATL).

Purification Step	Protein (mg)	Total Activity (Units)	Recovery (%)
Extract	12,000	307,200	100
Ammonium sulfate precipitation			
20% saturation		29,593	10
40% saturation		204,800	67
Hydrophobic chromatography	500	196,608	64
Ion-exchange chromatography			
Fraction A	240	10,752	4
Fraction B (ATL)	60	210,944	69

### 2.2. Hemagglutination Activity of ATL

**Figure 4:**
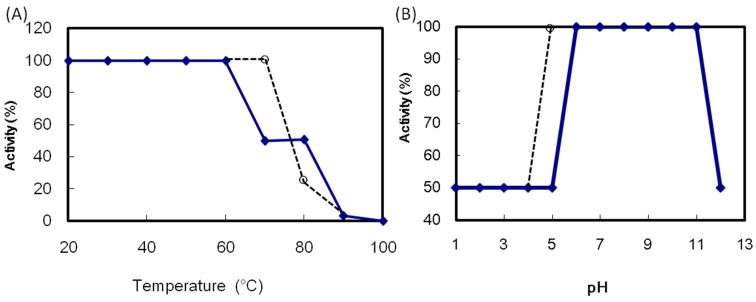
Effect of temperature and pH on the hemagglutination activity of *Apios* tuber lectin (ATL). (**A**) ATL was incubated at the indicated temperatures for 30 min; (**B**) ATL was incubated at various pH values overnight. After adjusting the pH to 7, the hemagglutination activity was measured. Solid line: ATL, broken line: Soybean lectin (SBA).

ATL maintained its maximum hemagglutination activity after incubation at 60 °C for 30 min (Figure 4A). The activity decreased above 70 °C and was lost after heating at 100 °C for 30 min. The ATL activity in the pH 6.0 to 11.0 was maintained (Figure 4B). The thermal and pH stability of ATL was comparable to those of SBA. The ATL hemagglutination activity was decreased by 75% after EDTA treatment (Table 2). The addition of Ca^2+^ led to the recovery of the maximum activity, indicating the requirement of Ca^2+^ for the hemagglutination activity of ATL. On the other hands, Mg^2+^ did not affect the activity. It is known that legume lectins are metalloproteins which contain Mn^2+^ and Ca^2+^ ion. Both divalent cations are required for the functional conformation of the monosaccharide binding site [2]. The present result suggests that ALT does not require Mn^2+^ for the activity but require Ca^2+^ to express full activity.

**Table 2:** Requirement of divalent metal ions for hemagglutination activity. The activity recovered was indicated when the non-treated activity was 100%.

Metal Ion (10 mM)	EDTA	Activity (%)
(−)	(−)	100
(−)	(+)	25
Ca^2+^, Mg^2+^	(+)	100
Ca^2+^	(+)	100
Mg^2+^	(+)	25

EDTA: ATL was treated with 0.1 M EDTA.

### 2.3. N-Terminal Sequencing of Proteins Contained in Apios Tubers

Five major and a few minor bands could be detected when the extract of *Apios* tubers was subjected to SDS-PAGE (Figure 2, lane E). The N-terminal amino acid sequence of each protein band was analyzed by Edman degradation on a gas-phase protein sequencer after electroblotting onto a PVDF membrane. The N-terminal amino acid sequences of bands (a), (b), and (d) were EDNNELQNYVPVYVMLPLE, ERLNPGDIYVPVYVMLPLEL, and NPVLDMDGDLVQNGGAYYILPVIRGKGGGIERAVTGKETTPLYTVVQS, respectively. They are similar to those of β-amylase from soybean (85% homology), β-amylase from *Arabidopsis thaliana* (80% homology), and Kunitz type trypsin inhibitor from soybean (72% homology), respectively. This is the first time that the presence of β-amylase- and Kunitz type trypsin inhibitor-like protein are shown in *Apios* tubers, though the Bowman-Birk type trypsin inhibitor, corresponding to band e, has been reported by Zhang *et al.* [20,21].

**Figure 5:**
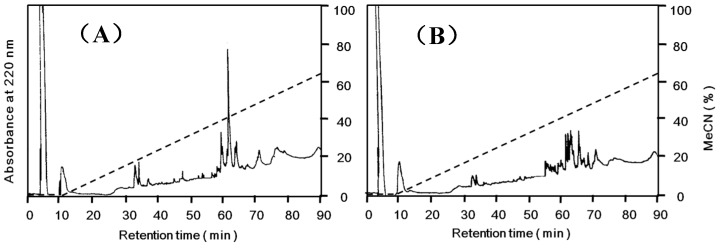
Peptide maps of *Apios* tuber lectin (ATL) and Fraction A. Each protein was reduced and *S*-carboxamidomethylated, and digested with arginylendpeptidase. Peptides were separated by reversed-phase HPLC on TSKgel ODS 120T (5 μm, 4.6 × 250 mm) using a linear gradient of acetonitrile in 0.1% trifluoroacetic acid at a flow rate of 1.0 mL/min. (**A**) ATL; (**B**) Fraction A.

The first 30 amino acid residues of band (c) containing ATL and Fraction A protein were determined to be AKLPFFSFNLDRFFPNEPNLIFQGDAKASS, which was completely consistent with the deduced *N*-terminal amino acid sequence of *Apios americana* lectin (AAL) [22]. Both ATL from SDS-PAGE and Fraction A protein separated by ion-exchange chromatography also showed the same *N*-terminal amino acid sequences, however, they gave slightly different peptide maps (Figure 5).

**Figure 6:**
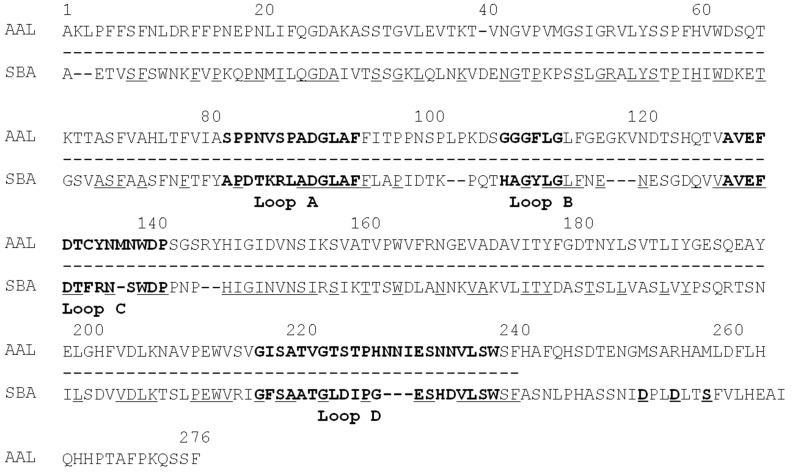
Sequence comparison of *Apios* tuber lectin (AAL) with soybean lectin (SBA). Identical amino acid residues are underlined beneath the sequence of SBA. The broken lines under the sequence of AAL are the sequence which was confirmed by direct sequencing of ATL using a protein sequencer in this study. Loops A-D are indicated by boldface letters. Asp (D) 240, Asp (D) 243, and Ser (S) 246 are putative truncating sites of SBA [24]. The amino acid sequence of AAL deduced from cDNA sequencing is cited from reference [22].

The amino acid sequence of *Apios* tuber lectin (ATL) was determined by direct protein sequencing of peptides generated by digestion of *S*-carboxamidomethylated (CAM)-ATL with several proteases, *Achromobacter* lysyl-endopeptidase, *Staphylococcis aurous* V8 protease, and mouse submandibular arginylendopeptidase. The digests were separated by RP-HPLC on a TSKgel ODS 120T column (4.6 × 250 mm) using a linear gradient of acetonitrile in 0.1% trifluoroacetic acid. The peptide fragments were subjected to protein sequencing by a protein sequencer and MALDI-TOF-MS spectrometry. The amino acid sequence of ATL (the sequences of the fragments are not shown) was consisted with the deduced amino acid sequence of *Apios* tuber lectin (AAL) cDNA reported by Kouzuma *et al.* [22], though the *C*-terminal amino acid sequence of ATL could not be determined in this study, due to the absence of the corresponding peptide fragments (Figure 6).

ATL showed a significant sequence homology to other legume lectins, such as soybean lectin (SBA), *Concanavalin* A (ConA), and *Ulex europeus* agglutinin-II (UEA-II), with 30%–40% amino acid identity. The legume lectins show a characteristic four loop structures, *i.e.*, loops A, B, C and D, which are conserved among legume lectins and might be related to carbohydrate-binding properties. Loop D seems to be correlated with carbohydrate-binding specificity [23]. The loop D of mannose-binding lectins (ConA) consists of 18 amino acid residues, whereas that of *N*-acetyl galactosamine-binding lectins (SBA and UEA-II) consists of more than 18–19 amino acid residues. ATL, showing glucosamine- and galactosamine-binding activity, has 23 amino acid residues in loop D. These results demonstrate that the characteristic loop structures of the legume lectin family allow the formation of binding sites with a wide range of specificities.

Ion-exchange chromatography (Figure 1B) revealed that the proteins contained in band c (Figure 2) consisted of two protein fractions. They showed the same *N*-terminal amino acid sequences and the same subunit structures, but different hemagglutination activities and different peptide maps. The ratio of the contents of *Apios* tube lectin (ATL) and non-lectin component (Fraction A protein) contained in the fractions was 1:4. Kouzuma *et al.* [22] reported the purification and cDNA cloning of *Apios* tuber lectin, named AAL. AAL required the minimal concentration of 125 μg/mL to agglutinate rabbit erythrocytes and the hemagglutinating activity was not inhibited by any monosaccharide. On the other hand, ATL isolated in this study required the minimal concentration of 4μg/mL to agglutinate the erythrocytes and the hemagglutinating activity was inhibited by 63 mM d-glucosamine and d-galactosamine. Furthermore, the molecular mass of AAL was estimated to be 30,110 Da by its cDNA sequence and SDS-PAGE, while for ATL a value of 28,400 Da was obtained by MALDI-TOF mass spectrometry. We do not have experimental results to explain these discrepancies between AAL and ATL. Since the major part of the amino acid sequence deduced by cDNA sequencing could be confirmed in this study, the lack of the C-terminal part of ATL may result in the smaller molecular mass. In fact, we could not find the peptide fragments corresponding to the *C*-terminal part from the proteolytic digests prepared in this study. One plausible explanation is the truncation of AAL to ATL at the *C*-terminal segment. Legume lectins are generally synthesized as a precursor, which subsequently is post-translationally processed into a mature protein. This post-translational processing may consist of proteolytic cleavage of the precursor chain, *C*-terminal trimming, removal of covalent carbohydrate, and even ligation of the original *C*- and *N*-termini [25]. SBA, *Phaseolus vulgaris* lectin (PHA-E), and the lectins from *Dolichos biflorus* have been known to contain ragged *C*-terminal ends. SBA consisted of at least five isolectins. The subunits of the isolectins, SBA-I, SBA-II, and SBA-III, consisted of 240, 243, 246 amino acid residues. They were generated by cleaving at Asp240, Asp243, and Ser246, respectively, of the intact SBA consisting of 253 amino acid residues [25].

In addition, the presence of the protein corresponding to the Fraction A protein has not been reported in *Apios* tubers. The lack of hemagglutinating activity of Fraction A protein might be due to the different internal amino acid sequence as indicated by peptide mapping (Figure 5), because the substitution of an amino acid such as a cysteine residue sometimes abolishes the activity even in the case of legume lectins [22]. Kouzuma *et al.* [22] also reported the purification and cDNA cloning of a lectin-like protein (AALP) from *Apios* tubers as a minor side fraction of AAL. AALP had no hemagglutinating activity with a homologous amino acid sequence to AAL. However, the Fraction A protein is a distinct molecule from AALP, because the *N*-terminal amino acid sequences (ADSLSFSFKEFTADPEDLIF) and molecular mass (26,305 Da) of AALP are quite different from those of Fraction A protein. Taken together, the discrepancy between the characteristics of ATL and AAL might be caused by the truncation of AAL to ATL at the *C*-terminal segment.

### 2.4. Sugar Binding Specificity

The sugar binding specificity of ATL was examined by the hemagglutination inhibition assay with rabbit erythrocytes (Table 3). The hemagglutination activity of ATL was inhibited by rather high concentrations of d-galactosamine and d-glucosamine at 63 mM, d-galactose (Gal) and d-ribose at 125 mM, d-mannose (Man) at 250 mM, and d-glucose (Glc), Me-α-d-mannopyranoside, and l-fucose (Fuc) at 500 mM. No inhibition was observed with *N*-Ac-glucosamine (GlcNAc) and *N*-Ac-galactosamine (GalNAc) even at a concentration of 1.0 M.

**Figure 7:**
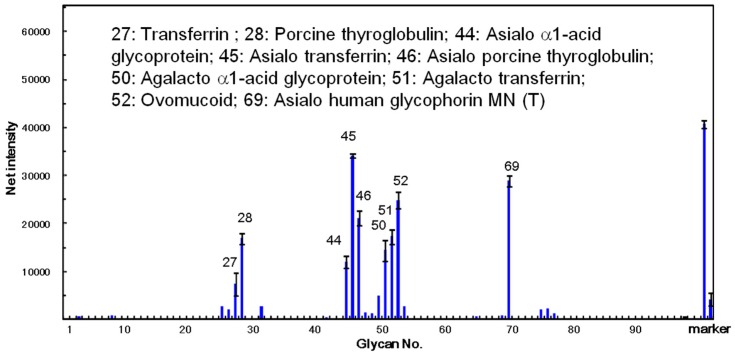
Specificity profiling of *Apios* tuber lectin (ATL). Scan images of ATL (12 ng/well) were analyzed with the Array Pro analyzer ver. 4.5. The net intensity value for each spot was determined as the signal intensity minus the background value. Data are the average ± S.D. of triplicate determinations. Detailed information on the glycans can be found in reference [26].

The carbohydrate-binding specificity of ATL was investigated using glycoconjugate microarray assays where 96 glycoconjugates were analyzed at the same time. The fluorescent image data analyzed with the Array Pro analyzer Ver. 4.5 (Media Cybernetics, Inc., Rockville, MD, USA), are shown in Figure 7. The results clearly demonstrated that ATL mainly bound to the asparagine (*N*)-linked sugar chain including its desialylated and agalactosylated glycoproteins, such as α1-acid glycoprotein, transferrin, and glycophorin MN (T). The lectin also showed high affinity against ovomucoid. These results indicate that ATL has a high binding activity against glycoproteins containing various complex sugar chains.

**Table 3:** Inhibition of hemagglutination activity of ATL by saccharides.

Sugar ^a^	mM
d-Galactosamine	63
d-Glucosamine	63
d-Galactose	125
d-Ribose	125
d-Mannose	250
d-Glucose	500
d-Me-α-d-mannopyranoside	500
L-Fucose	500
*N*-Ac-d-galactosamine	>1000
*N*-Ac-d-glucosamine	>1000

^a^ Minimum concentration of saccharides required for complete inhibition.

Many legume lectins contain, in addition to their carbohydrate binding site, one or more binding sites for hydrophobic ligands such as adenine and adenine-related plant hormone [24]. The broad binding specificity of legume lectins is explained by the fact that substitutions of amino acids involved in sugar binding activity and variations in the length of a particular loop, profoundly change the structure of the binding site without affecting the overall three-dimensional structure of lectin [27]. The interaction with simple sugars takes place in the monosaccharide-binding site located at the surface of the lectin. On the other hand, the oligosaccharides of glycoproteins interact with the extended carbohydrate-binding site comprising a number of adjacent residues beside a central monosaccharide-binding site. This characteristic feature of the binding sites may be correlated with the unique binding specificity of ATL.

### 2.5. Effect of ATL on Caco-2 Cell Monolayers

**Figure 8:**
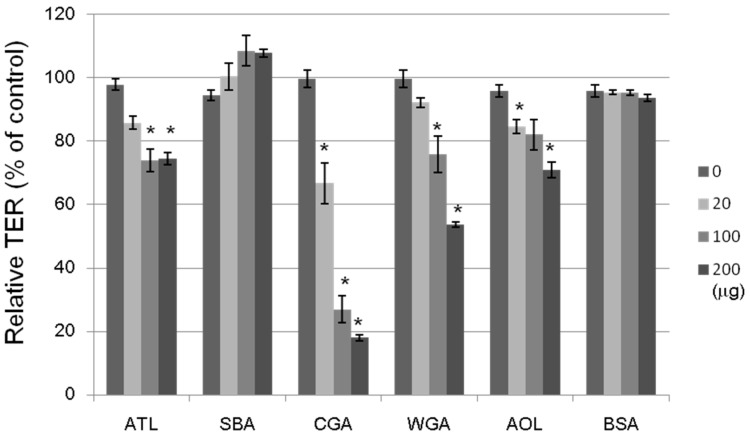
Effects of lectins on the TER values of Caco-2 monolayers. The cell monolayer TER value was measured after incubating for 2 h with a lectin (20–200 μg/mL). ATL: *Apios* tuber lectin, SBA: Soybean lectin, CGA: Japanese jack bean lectin, WGA: Wheat germ lectin, and AOL: *Aspergillus oryzae* lectin. Bovine serum albumin (BSA) (20–200 μg/mL) was used as a reference. Results are expressed as the percentage of the control value without a lectin at 0 h, and are the mean ± S.D. of three different determinations. * *p* < 0.05, compared with the control value.

The tightness of the intercellular junctions was evaluated by transepithelial electrical resistance (TER) measurements; a decrease in TER would indicate an increase in paracellular transport, or vice versa (Figure 8). The non-lectin protein, bovine serum albumin (BSA; 20–200 μg/mL), had no effect on the TER value. The ATL, Japanese jack bean lectin (CGA), wheat germ lectin (WGA), and *Aspergillus oryzae* lectin (AOL) treatments at 100 μg/mL each decreased the TER value by 20%–70% after a 2-h incubation period, whereas SBA treatment did not change at all. We investigated the effects of 16 lectins with varying sugar-binding specificities on the transport system across Caco-2 cell monolayers by using four fluorescent markers, whose transport pathways were known. ATL decreased the TER value, indicating an increase in paracellular transport. It has been also shown that other lectins having distinct sugar binding specificities: e.g., CGA for Man, WGA for GlnNAc, and AOL for L-fucose (Fuc) exhibited similar effects. Although the precise mechanisms underlying these modulating effects remain unclear, lectins may modulate the transport system through intracellular signaling, controlling the expression of various proteins, or assembling and disassembling cytoskeletal proteins, as has been reported for other food components.

The absorption of nutrients and food factors across the intestinal epithelium occurs because of one or more different transport pathways such as passive paracellular transport, passive transcellular transport, and carrier-mediated transport. We have shown that several lectins, including SBA, CGA, and WGA, increased the transport of isoflavones but not their aglycones. The TER across a Caco-2 cell monolayer reflects any effects on the TJ-mediated paracellular pathways. ATL, which decreased the TER, may have any effect on the intestinal transport system if its active form can reach the intestine.

## 3. Experimental Section

### 3.1. Materials

*Apios* tubers were obtained from Tohoku Tenma Agricultural Cooperative, Aomori Prefecture, Japan, and stored at 4 °C until use. Toyopearl phenyl-650M, Toyopearl DEAE-650M, TSKgel ODS 120T (5 μm, 4.6 × 250 mm) were purchased from Tosoh (Tokyo, Japan). *Aspergillus oryzae* lectin (AOL) was kindly supplied by Gekkeikan (Koyto, Japan) [28]. SBA, Japanese jack bean lectin (CGA), and wheat germ lectin (WGA) were isolated as previously described [12]. A human colon adenocarcinoma cell line, Caco-2 cells, was obtained from the American Type Culture Collection (Rockville, MD, USA). Dulbecco’s modified eagle’s medium (DMEM), non-essential amino acid (NEAA), penicillin-streptomycin (10,000 units/mL and 10 mg/mL in 0.9% sodium chloride), and *N*-[2-hydroxyethyl] piperazine-*N'*-[2-ethanesulfonic acid] (HEPES) were purchased from Sigma (St. Louis, MO, USA). Plastic dishes and 96-well micro-titer plates were purchased from Becton Dickinson and Company (Franklin Lakes, NJ, USA). All other chemicals used in this study were of analytical grade.

### 3.2. Isolation of Apios Tuber Lectin (ATL)

*Apios* tubers (200 g) were homogenized with 20 mM Tris-HCl buffer (pH 8.0, 600 mL) and centrifuged at 12,000× *g* for 30 min at 4 °C. The supernatant was mixed with solid ammonium sulfate to 20% saturation, left for 1 h at 4 °C, and centrifuged at 12,000× *g* for 30 min at 4 °C. Solid ammonium sulfate was added to the obtained supernatant to 40% saturation, left for 1 h at 4 °C, and centrifuged at 12,000× *g* for 30 min at 4 °C. The precipitate was dissolved in 50 mM Tris-HCl buffer (pH 8.0, 100 mL).

The lectin solution was subjected to hydrophobic chromatography on a Toyopeal phenyl-650M column (2.8 × 16 cm) pre-equilibrated with 1 M ammonium sulfate in 50 mM Tris-HCl buffer (pH 8.0). The column was developed with a decreasing linear gradient of ammonium sulfate (1.0–0 M) in 50 mM Tris-HCl (pH 8.0). Fractions (5 mL/tube) were collected, and the absorbance at 280 nm was recorded. The fractions exhibiting lectin activity were pooled, and dialyzed against 50 mM Tris-HCl (pH 8.0).

The dialysate was subjected to anion exchange chromatography on a Toyopearl DEAE-650M column (2.8 × 32 cm) pre-equilibrated with 50 mM Tris-HCl (pH 8.0) and eluted with a linear gradient of NaCl (0 to 1.0 M) in the same buffer. The fractions exhibiting lectin activity were pooled, dialyzed against distilled water, and lyophilized to give *Apios* tuber lectin (ATL).

### 3.3. Hemagglutination Assay and Inhibition Assay

Samples were diluted 2-fold (v/v) in series with 0.15 M NaCl (50 μL) containing 20 mM CaCl_2_ in 96-well microtiter plates, and mixed with 2% rabbit erythrocyte suspension (50 μL). The mixture was allowed to stand at room temperature for 60 min, and then the hemagglutination activity was measured. The titer value was defined as the maximum dilution with positive agglutination of rabbit erythrocytes. The thermostability of ATL was examined by the hemagglutination assay described above after separate incubations at 20 °C, 30 °C, 40 °C, 50 °C, 60 °C, 70 °C, 80 °C, 90 °C and 100 °C for 30 min.

ATL (0.5 mg/mL) was incubated at various pH values overnight at 4 °C. The buffers used were 50 mM HCl-KCl containing 0.15 M NaCl (pH 1.0 to 2.0), 50 mM CH_3_COONa containing 0.15 M NaCl (pH 3.0 to 6.0), 50 mM Tris-HCl containing 0.15 M NaCl (pH 7.0 to 9.0), 50 mM sodium tetraborate containing 0.15 M NaCl (pH 10.0), and 50 mM NaCl-KCl containing 0.15 M NaCl (pH 12.0). After adjusting the pH to 7.0 with 1 M HCl or 1 M NaOH, hemagglutination activities were measured.

To test the dependence of divalent cations on hemagglutination, ATL was treated with 0.1 M EDTA in 20 mM Tris-HCl (pH 8.0) containing 0.15 M NaCl at room temperature for 2 h, and then dialyzed against the buffer overnight at 4 °C. The lectin solution was tested for hemagglutination activity in the absence or presence of Ca^2+^ or Mg^2+^ in 20 mM Tris-HCl (pH 8.0) containing 0.15 M NaCl.

### 3.4. Molecular Mass Determination of ATL

The molecular mass of ATL and the subunit structure were determined by sodium dodecyl sulfate-polyacrylamide gel electrophoresis (SDS-PAGE), size exclusion chromatography, and matrix-assisted laser desorption ionization time of flight (MALDI-TOF) mass spectrometry (Voyager-DE^TM^ STR, Applied Biosystems, Foster City, CA, USA).

SDS-PAGE was performed according to the method of Laemmli [29] using 15% separating gel in the presence or absence of 2-mercaptoethanol, and protein bands were stained by Coomassie Brilliant Blue R-250. The protein markers used were bovine serum albumin (66 kDa), egg white albumin (45 kDa), carbonic anhydrase (29 kDa), myoglobin (17 kDa), and cytochrome C (12.5 kDa). Size exclusion chromatography was performed by using a PC200S (N) column of phosphorylcholine immobilized on silica gel beads (5 μm) (7.8 × 300 nm, Shiseido, Tokyo, Japan) and 50 mM HEPES containing 0.25 M NaCl and 5 mM CaCl_2_ as the mobile phase [30]. The MALDI-TOF mass spectrum was measured by a linear mode with an acceleration voltage of 25 kV using sinapinic acid as the matrix. Insulin (5733.5 Da) (bovine pancreas) and myoglobin (16,949.5 Da) (horse heart) were used as standards for external calibration.

### 3.5. N-Terminal Sequencing of Proteins Contained in Apios Tubers

The extract of *Apios* tubers was separated by SDS-PAGE and blotted onto polyvinylidene difluoride (PVDF)-membranes at 0.1 volt for 60 min. The protein bands were stained with Coomassie Brilliant Blue R-250, cut carefully and washed with ethanol and distilled water thoroughly to remove the dye and salts. Each small piece of PVDF membrane, representing a band, was subjected to sequencing by a protein sequencer (PPSQ-10, Shimadzu, Kyoto, Japan).

ATL (1.0 mg) was reduced and *S*-carboxamidomethylated (CAM). CAM-ATL was digested with endoprotinase Lys-C (substrate/enzyme (S/E), 100:1), *Staphylococcus aureus* V8 protease (S/E, 50:1), and endoproteinase Arg-C (S/E, 100:1). Each digest was separated by reversed-phase high-performance liquid chromatography (RP-HPLC) on a TSKgel ODS 120T column using a linear gradient of acetonitrile in 0.1% trifluoroacetic acid. The amino acid sequences of the isolated peptide fragments were determined by the combined use of a protein sequencer and MALDI-TOF mass spectrometer. Homologous sequences were searched by the BLAST program and FASTA program accessed by NCBI. Multiple sequence alignment was performed by the ClustalW program accessed by DDBJ.

### 3.6. Sugar Binding Specificity

The inhibitory effects of saccharides on hemagglutination were assayed as follows. The saccharide solutions (25 μL) were diluted 2-fold in series in 96-well microtiter plates and incubated with 25 μL of the lectin solution of hemagglutination titer values of 2^−3^ for 15 min. The rabbit erythrocytes suspension (2%, 50 μL) was added to the mixture and incubated for another 30 min. The inhibitory activities were estimated by the minimum concentration of sugar needed to cause negative hemagglutination.

### 3.7. Glycoconjugate Microarray Assay

Glycoconjugate microarray assay was carried out as described [26]. Glycoproteins and glycoside-polyacrylamide (PAA) conjugates were immobilized on a microarray-grade epoxy-coated glass slide (Schott AG, Mainz, Germany). Cy3-labeled ATL precomplexed with Cy3-labeled antibodies in the probing buffer (25 mM Tris-HCl, pH 7.4 containing 0.8% NaCl, 1% (v/v) Triton-X, 1 mM MnCl_2_, 1 mM CaCl_2_) was applied to each chamber of the glass slides (100 μL/well) and incubated at 20 °C overnight. Fluorescent images were immediately acquired using an evanescent-field activated fluorescence scanner (SC-Profiler, Moritex, Tokyo, Japan) under Cy3 mode without washing steps. Throughout the experiments, the scanning conditions of the cooled charge-coupled device (CCD) camera, *i.e.*, resolution (5 μm), number of times for integration (4), and exposure time (200 sec), were fixed. Data were analyzed with the Array Pro analyzer Ver. 4.5 (Media Cybernetics, Inc., Rockville, MD, USA). The net intensity value for each spot was determined by the signal intensity minus the background value. Data are the average ± S.D. of triplicate determinations.

### 3.8. Cell Culture and TER Measurement

The Caco-2 human colon adenocarcinoma cell line was obtained from the American Type Culture Collection. Cells at passage numbers 23–35 were cultured in DMEM with 10% (v/v) FBS, penicillin-streptomycin (50 IU/mL and 50 μg/mL, respectively), and 1% (v/v) NEAA, and maintained at 37 °C in a humidified atmosphere of 5% CO_2_ in air. The cells were sub-cultured at 70%–80% confluency. Caco-2 cell monolayers were prepared by seeding on Transwell inserts with a 0.40-μm polycarbonate membrane 6.5 mm in diameter (Corning Costar, Rochester, NY, USA) at a density of 1.0 × 10^5^ cells/cm^2^. The apical and basolateral compartments contained 0.1 and 0.6 mL of the culture medium, respectively. The cell monolayers were maintained for 18–21 days (the culture medium was replaced every 2–3 days), and the integrity of the cell monolayers was evaluated by measuring the TER value with a Millicell-ERS instrument (Millipore, Billerica, MA, USA). Cell monolayers with TER values of >500 Ω/cm^2^ were used for subsequent experiments.

The lectins were dissolved in HBSS containing 0.8 mM *N*-(2-hydroxyethyl) piperazine-*N'*-2-ethanesulfonic acid (HEPES, at pH 7.3). The cell monolayers were gently rinsed twice with HBSS, and then treated for 2 h with each lectin (20–200 μg/mL). The TER value of the monolayers was measured before and after the treatment.

## 4. Conclusions

*Apios* tuber lectin isolated from *Apios americana* Medikus showed binding affinity against several monosaccharides, such as d-glucosamine and d-galactosamine, and desialylated or agalactosylated glycoproteins such as asialo and agalacto transferrin. The lectin decreased the transepitherial electrical resistance value across human intestinal Caco-2 cell monolayers, suggesting the effect on the tight junction-mediated paracellular transport.
